# Successful long‐term guselkumab treatment of severe plaque psoriasis in patients with class III obesity: A case series

**DOI:** 10.1002/ski2.289

**Published:** 2023-09-19

**Authors:** Marco Galluzzo, Lorenzo Marcelli, Angela Fico, Luca Bianchi, Marina Talamonti

**Affiliations:** ^1^ Department of Systems Medicine University of Rome “Tor Vergata” Rome Italy; ^2^ Dermatology Unit Fondazione Policlinico “Tor Vergata” Rome Italy

## Abstract

Data from real‐world studies and clinical trials have documented the long‐term efficacy and safety of guselkumab in patients with moderate‐to‐severe psoriasis. Limited data are available on the long‐term use of guselkumab in morbidly obese individuals with severe psoriasis. Here, we present data on the outcome of three patients with class III obesity (body mass index (BMI) of ≥40 kg/m^2^) with severe plaque psoriasis treated with 100 mg guselkumab. At baseline, mean BMI was 46.5 ± 5.4 kg/m^2^ and mean PASI was 46.0 ± 18.5 and all patients were biologic naïve. After 12 weeks of guselkumab treatment, mean PASI decreased to 9.7 ± 4 and to 4.0 ± 1.7 at 28 weeks. After 1 year, two patients achieved complete remission and one patient had PASI of 6 (achieving remission by week 140). All three patients are still in complete remission. Our real‐life results in specific patients burdened with class III obesity naïve to biologic treatment show excellent long‐term psoriasis outcome with guselkumab.

## Graphical abstract

Patients with severe plaque psoriasis who are morbidly obese and naive to biological treatment may benefit from long‐term treatment with guselkumab.

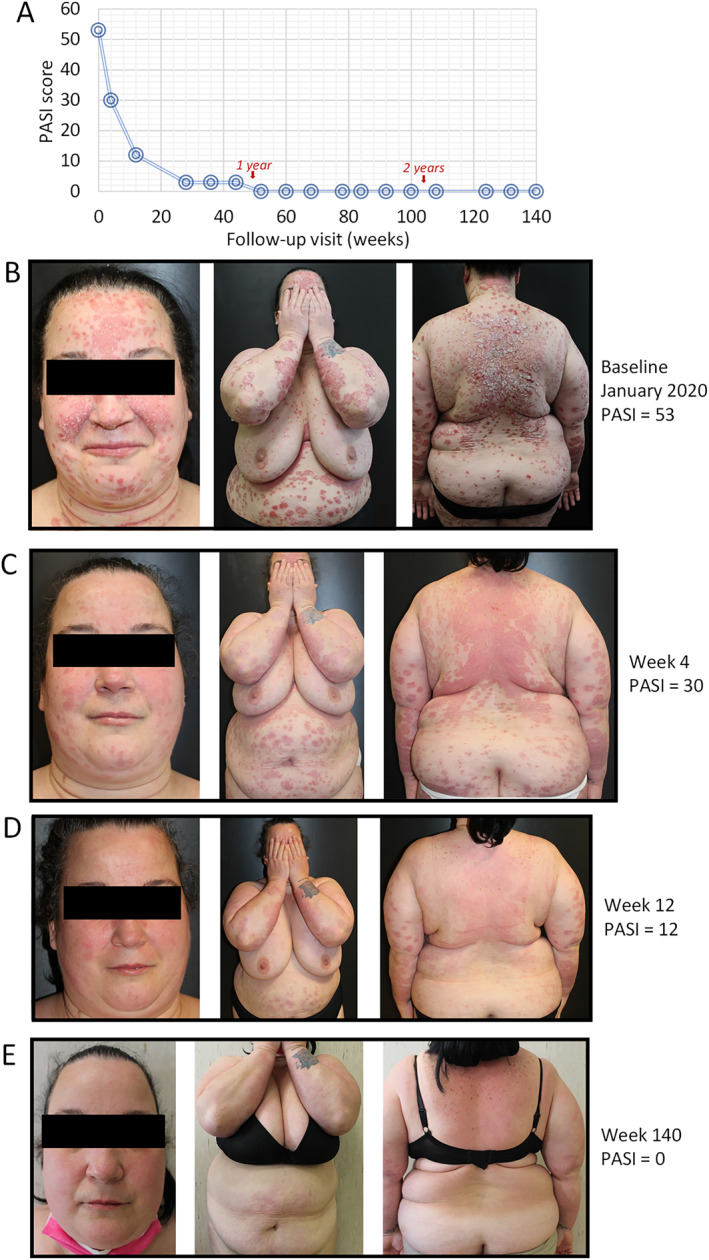

## 1 INTRODUCTION

Data from studies undertaken in the real‐word setting show that biological drugs are associated with partial response or lack of efficacy in patients with excess adipose tissue.^1^, ^2^ Although various studies have demonstrated that the efficacy of biological therapies can be influenced by obesity,^1^, ^2^ sub‐analyses of randomized clinical trials evaluating the impact of body mass index (BMI) on the efficacy of biological therapies have shown conflicting results.^3^, ^4^ Real‐life studies have mainly considered patients treated with anti‐tumour necrosis factor‐alpha (TNF‐alpha) or anti‐interleukin (IL)‐12/23 drugs, while few studies have examined the effectiveness of novel IL‐23 biologics in this setting.

Guselkumab is an anti‐IL‐23 biologic therapy approved in 2017 for the treatment of adult patients with moderate‐to‐severe psoriasis. Data from clinical trials of guselkumab in patients with moderate‐to‐severe psoriasis have documented that response rates are lower in patients with either higher body weight (≥90 kg)^5^ or obesity (BMI of ≥30 kg/m^2^).^6^ Although some recent real‐world studies have assessed the impact of BMI on the effectiveness of guselkumab in patients with moderate‐to‐severe plaque psoriasis,^7^, ^8^, ^9^, ^10^, ^11^, ^12^ the impact of high BMI on the real‐world effectiveness of guselkumab remains an important area of interest, particularly in cases with severe difficult‐treat plaque psoriasis. In this case series we present findings from three patients with class III obesity (BMI ≥40 kg/m^2^) and severe psoriasis (PASI score of 25–60) treated with guselkumab.

## 2 CASE DESCRIPTION

### 2.1 Case 1

**FIGURE 1:**
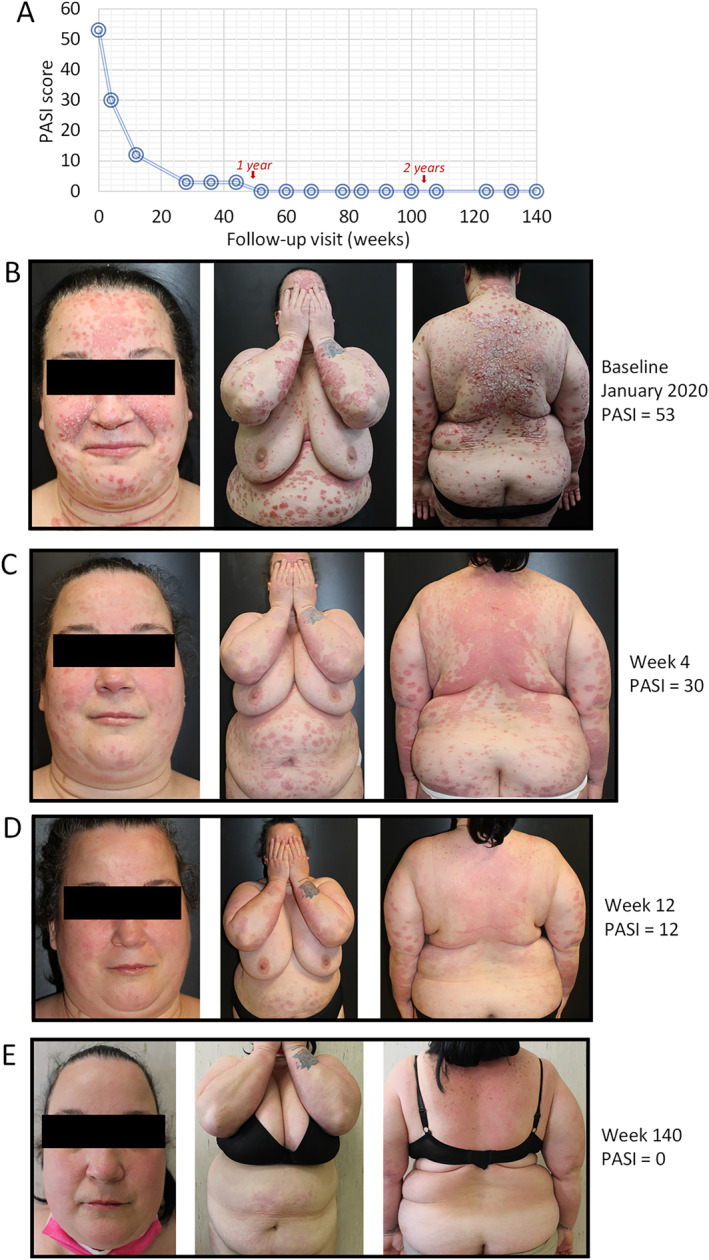
Representative images of outcome in Case #1; a female patient, aged 46 years with onset of psoriasis at 39 years of age and naïve to biological treatment. In January 2020 she presented with severe plaque psoriasis and was morbidly obese (body mass index (BMI) of 43 kg/m^2^). The change in PASI score is presented in Figure 1a. After baseline visit (PASI of 53), guselkumab treatment was initiated (induction phase: 100 mg subcutaneously at weeks 0 and 4, and a maintenance dose every 8 weeks thereafter) (Figure 1b). After 4 weeks, PASI decreased to 30 (Figure 1c) and after 12 weeks PASI decreased to 12 (Figure 1d) and at week 140, PASI decreased to 0 (Figure 1e).

The first case involved a 46‐year‐old female patient who presented in our clinic in January 2020 with severe widespread chronic plaque psoriasis since the age of 39 (Figure 1). She was a non‐smoker, consumed alcohol occasionally and denied taking any current medication. She weighed 110 Kg, height of 1.6 m and had a BMI of 43 kg/m^2^. She was previously treated with (and non‐responsive) to methotrexate and naïve to biological drugs. Only mild hyperuricemia (uric acid of 7.1 mg/dL) was recorded on screening tests, that remained unchanged during follow‐up examinations. After baseline visit on 23/01/2020 (PASI of 53), guselkumab treatment was initiated (induction phase: 100 mg subcutaneously at weeks 0 and 4, and a maintenance dose every 8 weeks thereafter) (Figure 1b). After 4 weeks, PASI decreased to 30 (Figure 1c) and after 12 weeks PASI decreased to 12 (Figure 1d). In January 2021, following hyperglycemia (blood glucose of 129 mg/dL), the patient was sent for an endocrinological examination, diagnosed with type 2 diabetes and subsequently treated with metformin. In November 2021 there was a further increase in weight to 125 kg while still maintaining complete remission from the disease starting from week 52. Last PASI score recorded in October 2022 was 0 (on week 140, PASI 100 was maintained) (Figure 1e).

### 2.2 Case 2

**FIGURE 2:**
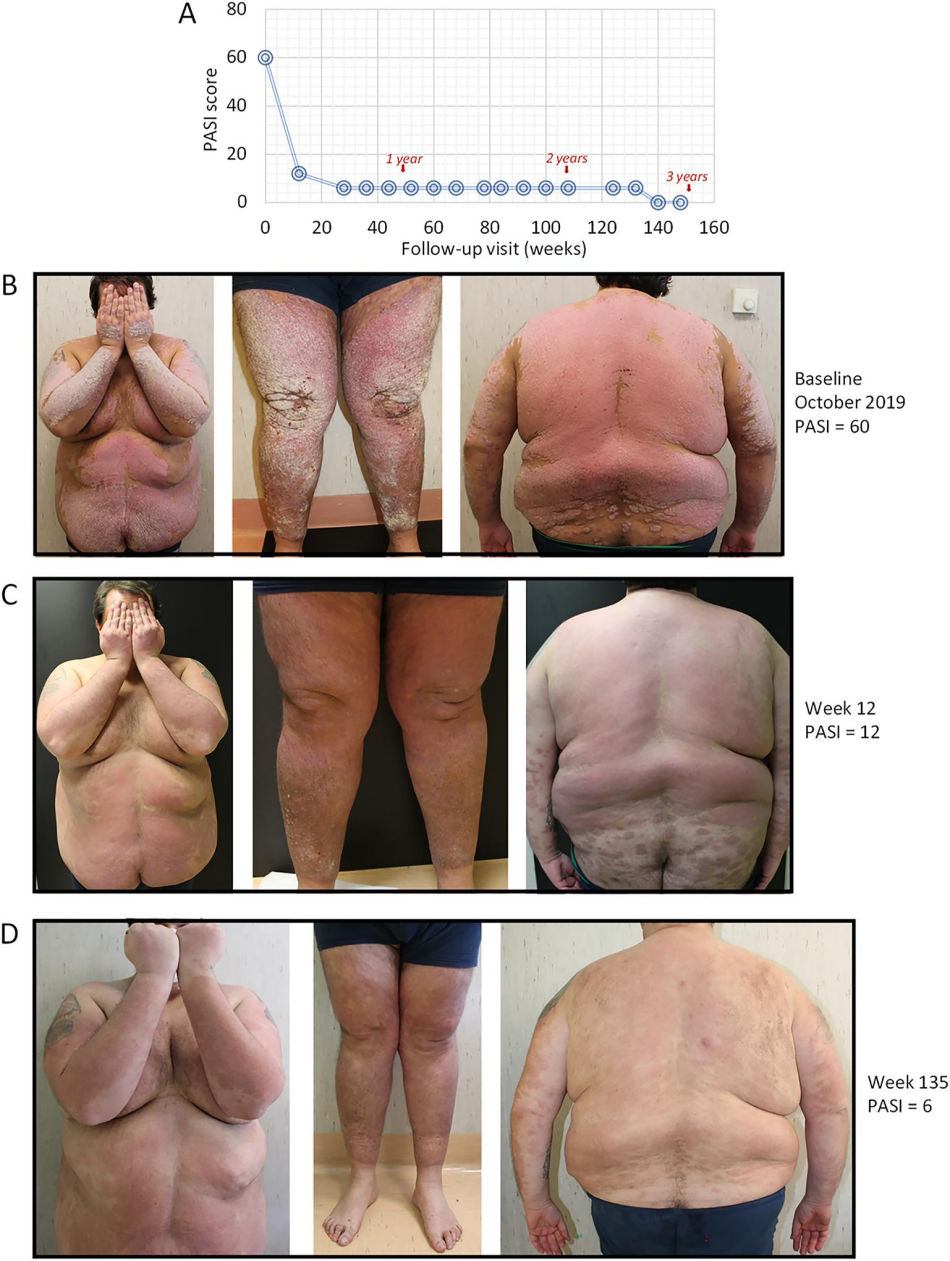
Representative images of outcome in Case #2; a male patient, aged 38 years with onset of psoriasis at 32 years of age and naïve to biological treatment. In October 2019 he presented with severe plaque psoriasis and was morbidly obese (body mass index (BMI) of 52.6 kg/m^2^). The change in PASI score is presented in Figure 2a. After baseline visit (PASI of 60), guselkumab treatment was initiated (induction phase: 100 mg subcutaneously at weeks 0 and 4, and a maintenance dose every 8 weeks thereafter) (Figure 1b). After 12 weeks PASI decreased to 12 (Figure 2c) and at week 135, PASI decreased to 6 (Figure 2d).

In October 2019 a 38‐year‐old male patient presented with severe chronic plaque psoriasis since the age of 32 years (Figure 2). He weighed 190 kg, height of 1.9 m and had a BMI of 52.6 kg/m^2^. He had prior failure to cyclosporine and was naïve to biological drugs. On 24/10/2019 guselkumab treatment (100 mg) was initiated and PASI decreased from 60 at baseline (Figure 2b) to 12 at week 12 and 6 at week 135 (Figure 2d). No adverse events or changes in laboratory parameters were observed during treatment. The last recorded PASI was 3 on 10/11/2022 (week 156; almost clear, PASI 90 reached).

### 2.3 Case 3

**FIGURE 3:**
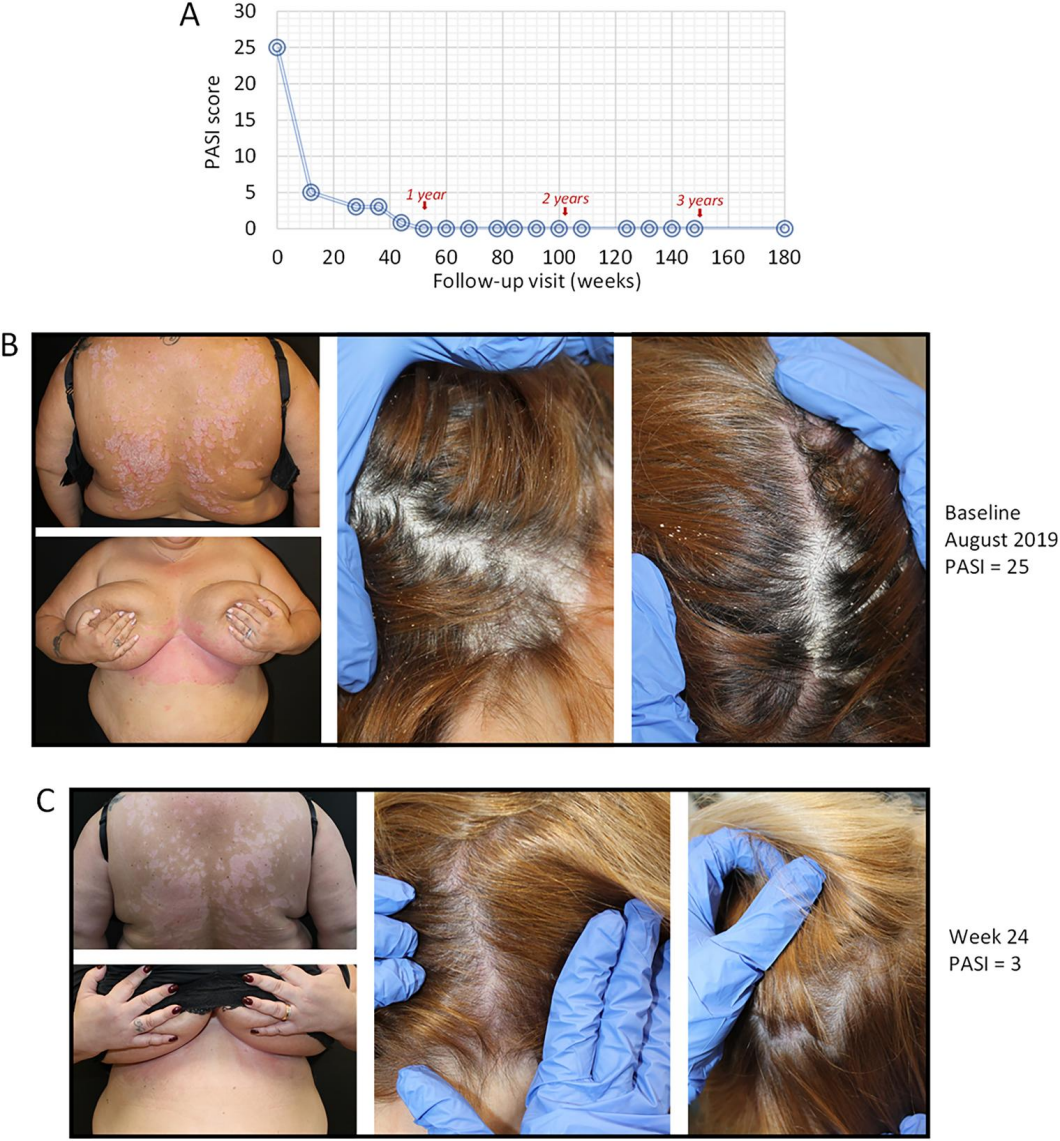
Representative images of outcome in Case #3; a female patient, aged 47 years with onset of psoriasis at 43 years of age and naïve to general systemic or biological treatment. In August 2019 she presented with severe plaque psoriasis and was morbidly obese (body mass index (BMI) of 52.6 kg/m^2^). The change in PASI score is presented in Figure 3a. After baseline visit (PASI of 25), guselkumab treatment was initiated (induction phase: 100 mg subcutaneously at weeks 0 and 4, and a maintenance dose every 8 weeks thereafter) (Figure 3b). After 24 weeks PASI decreased to 3 (Figure 3c).

The third case involved a 47‐year‐old female patient who presented with chronic plaque psoriasis since the age of 43 in addition to hypertension, dyslipidemia (triglycerides of 161 mg/dl and total cholesterol of 214 mg/dl) and severe obesity (weight = 112 kg; height = 1.60 and BMI = 43.8 kg/m^2^). As well as being affected mainly in regions of the lower back/shoulder, breast and genital region, this patient also presented with severe scalp involvement. The patient was previously treated with topical corticosteroids and vitamin D analogues and was naïve to general systemic as well as biological drugs. Treatment with guselkumab (100 mg) was started on 07/08/2019 (baseline PASI of 25; Figure 3b) and after 24 weeks PASI decreased to 3 (Figure 3c). The last PASI score recorded on 12/01/2023 was 0, at week 180 (complete clear skin, PASI 100 achieved). The patient achieved complete remission of disease starting from week 52 with resolution of scalp psoriasis starting from the 20th week of treatment. Furthermore, starting from the second year of treatment there was a progressive loss in body weight. The last recorded weight (on 12/01/2023) was 92 kg.

## 3 DISCUSSION

Data from this case series of three patients show that guselkumab given at the standard dosing regime of 100 mg at weeks 0 and 4, and then every 8 weeks thereafter was effective in achieving almost complete remission in all three patients as early as 4 months. Given that these patients were burdened with severe psoriasis (PASI ranging from 25 to 60 at baseline), and some had multiple comorbidities as well as being morbidly obese, further highlights the benefit afforded by guselkumab treatment.

We have previously evaluated the long‐term effectiveness of guselkumab in 122 patients with moderate‐to‐severe plaque psoriasis treated up to 148 weeks.^13^

The variable naive versus previous biological treatment emerged as a predictor during multivariate analysis associated with an improvement in PASI 75, 90 or 100 response by 28–100 weeks while BMI (<30 kg/m^2^ vs. ≥30 kg/m^2^) was not. It is important to note that the three patients examined in this case series were all biologic naïve, confirming the importance of this characteristic when treating with guselkumab.

Bardazzi et al. also showed that a decrease in PASI with guselkumab was not significantly influenced by the presence of ≥3 comorbidities, BMI >30 kg/m^2^, or a previous failure to ≥2 biologic therapies.^9^ Gargiulo et al., also reported similar effectiveness of guselkumab in obese and non‐obese patients with psoriasis in terms of PASI 75, 90, and 100 at week 104.^8^ Another observational study by Ricceri and colleagues evaluated the efficacy and safety of guselkumab (*N* = 56), risankizumab (*N* = 33) and tildrakizumab (*N* = 24) in overweight‐to‐obese patients with moderate to‐severe psoriasis up to 52 weeks.^7^ Although their analysis did not stratify by biologic type, they demonstrated that both efficacy and safety profiles were independent of patient body weight and anti‐IL‐23 agents showed sustained clinical benefit in the whole population, as well as in obese (*N* = 44) or overweight subcohorts (*N* = 69).

Armstrong recently evaluated the effectiveness of guselkumab by BMI category in patients with moderate‐to‐severe plaque psoriasis in the CorEvitas Psoriasis Registry.^14^ Their study included 180 patients and 101 (56%) were obese. They showed improvements in disease severity and several patient‐reported outcome scores within all BMI categories among patients with moderate‐to‐severe psoriasis treated with guselkumab. Their unadjusted findings suggest that obese and overweight patients have comparable absolute improvements to those with lower BMI.^14^

Taken together, findings from this case series and other real‐life studies suggest that it would be desirable that this particular class of patients (BMI ≥40) with moderate‐severe chronic plaque psoriasis be immediately started on therapy with an anti‐interleukin 23p19 (such as guselkumab) that can yield excellent control of the disease both in the short and long term. Considering that approximately one third of patients with moderate‐to‐severe psoriasis have obesity,^1^, ^5^, ^10^ the availability of biologics such as guselkumab offer significant benefit in this setting over several other classes of biologics (anti‐TNF, anti‐IL‐17A or IL‐12/23) where clinical response can be affected.^15^

## CONFLICT OF INTEREST STATEMENT

Marco Galluzzo and Marina Talamonti declare to have acted as speakers and/or consultants for AbbVie, Almirall, Eli‐Lilly, Janssen‐Cilag, LeoPharma, Novartis and Sanofi, outside the submitted work. Luca Bianchi declares to have acted as a speaker and consultant for AbbVie, Novartis, Janssen‐Cilag, Pfizer, UCB, and LeoPharma, outside the submitted work. The authors have no other relevant affiliations or financial involvement with any organization or entity with a financial interest in or financial conflict with the subject matter or materials discussed in the manuscript apart from those disclosed.

## AUTHOR CONTRIBUTIONS

**Marco Galluzzo**: Conceptualization (lead); formal analysis (equal); investigation (equal); methodology (equal); supervision (lead); visualization (lead); writing – original draft (equal); writing – review & editing (equal). **Lorenzo Marcelli**: Investigation (equal); writing – original draft (equal); writing – review & editing (equal). **Angela Fico**: Investigation (equal); writing – original draft (equal); writing – review & editing (equal). **Luca Bianchi**: Conceptualization (supporting); investigation (equal); supervision (supporting); writing – original draft (equal); writing – review & editing (equal). **Marina Talamonti**: Conceptualization (lead); formal analysis (equal); investigation (equal); supervision (lead); visualization (equal); writing – original draft (equal); writing – review & editing (equal).

## ETHICS STATEMENT

The study was conducted in accordance with the 1975 Declaration of Helsinki ethical standards. According to Italian law, formal ethical committee approval is not required for this type of study.

## Data Availability

All data relevant to patients described in this case series are presented in the paper. Additional patient data can be made available from the corresponding author upon reasonable request.
